# Impact of integrated quaternary ammonium compounds in conventional glass-ionomers on cariogenic oral microflora: An in vitro analysis

**DOI:** 10.34172/joddd.025.42583

**Published:** 2025-09-30

**Authors:** Aleksandar Dimkov, Jasna Simonoska, Elizabeta Gjorgievska

**Affiliations:** Department of Pediatric and Preventive Dentistry, Faculty of Dental Medicine, University “Ss Cyril and Methodius”, Skopje, North Macedonia

**Keywords:** Antimicrobial agents, Benzalkonium chloride, Cariogenic microorganisms, Cetylpyridinium chloride, Glass-ionomer cements

## Abstract

**Background.:**

In addition to the capacity for gradual and sustained fluoride release over extended durations, which is crucial for remineralization processes and antibacterial properties, glass-ionomer cements (GICs) may serve as templates for releasing additional active antimicrobial agents. This study aimed to evaluate and compare the antimicrobial activity of conventional GICs—ChemFlex and Fuji IX—with and without the addition of antimicrobial compounds benzalkonium chloride and cetylpyridinium chloride (CPC), against cariogenic bacteria *Streptococcus mutans*, *Lactobacillus casei*, and *Actinomyces viscosus* across different time intervals.

**Methods.:**

Specimens measuring 4×6 mm were prepared from the cements with and without the incorporation of antibacterial agents. The inhibitory zones were assessed after 48 hours, as well as after 2, 7, and 21 days of incubation. The agar diffusion method was employed to determine the zones of inhibition.

**Results.:**

The statistical analysis of the antimicrobial effects between the two compounds indicated no significant differences in the control group. Statistically significant differences were noted in the experimental group, except on the 21st day for *S. mutans* across all concentrations, and the 48th hour and 7th day for *A. viscosus* in 3% antimicrobial agents.

**Conclusion.:**

The incorporation of antimicrobial agents into conventional glass-ionomers demonstrated an inhibitory effect on all examined cariogenic bacteria. This effect was more significant at higher concentrations. Over time, the suppressive effect diminished; however, it remained significantly strong. Glass-ionomers lacking antimicrobial agents demonstrated a restricted but noticeable antibacterial efficacy.

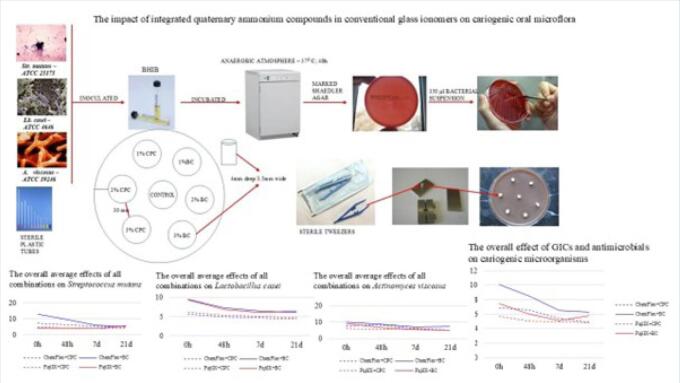

## Introduction

 While dental caries was previously viewed as a multifactorial disease, the latter part of the twentieth century and the early 21st century saw a predominant emphasis on its infectious characteristics. This conventional explanation posits that dental caries is an infectious condition leading to the deterioration of dental components, principally caused by bacterial contamination termed “infected dentin,” and demineralization identified as “affected dentin.” This necessitates the removal of the causative bacteria for effective therapy. Conversely, the modern interpretation of caries characterizes it as an ecological disruption within the dental biofilm, wherein acidogenic and aciduric bacteria become more competitive due to frequent carbohydrate consumption, leading to their predominance in the biofilm. Both definitions of dental caries identify microorganisms as a common “element.”^1-7^ Considering that bacterial flora is likely the most significant factor in the onset and progression of dental caries, as well as in the formation of secondary caries beneath restorations, initiatives to develop diverse preventive and restorative strategies aimed at reducing bacterial presence and consequently interrupting this cycle are justified.

 The efficacy of antimicrobial compounds in toothpastes, gels, or mouthwashes is well established; however, integrating these compounds into restorative materials and ensuring their sustained release in the oral cavity and dental tissues would offer additional advantages in eliminating cariogenic microorganisms.^8-10^

 Since their introduction to the restorative materials market in the early 1970s, glass-ionomer cements (GICs) have emerged as highly regarded restorative materials. They exhibit the beneficial properties of fluoride in remineralization processes, antimicrobial efficacy, biocompatibility, and similar thermal expansion coefficients to dental structures. Consequently, the World Health Organization (WHO) has included GICs in the WHO Model List of Essential Medicines since 2021, signifying the most effective, safe, and cost-efficient medications for priority illnesses.^11-13^ Despite several classifications and subcategories present in the literature, GICs are commonly categorized into five basic types: conventional GICs, resin-modified GICs, hybrid ionomer cements, resin-modified glass ionomers or dual-cured GICs, tri-cure GICs, and metal-reinforced GIC or Cermets.^14-16^ The composition and the chemical reactions of GICs are basically equal for all categories, with some variations in the powder/liquid ratio, as well as in the particle size, which is adapted for different purposes.^15,16^ In addition to their capacity for gradual and sustained fluoride release over extended durations, which is crucial for remineralization processes and antibacterial properties, GICs may serve as templates for releasing additional active antimicrobial agents.^16-20^ Research indicates that numerous efforts have been undertaken to include chlorhexidine in various formulations, concentrations, and combinations in other medicines, to identify the most effective delivery method for diminishing cariogenic bacteria in saliva and plaque.^19-22^ Although there is some knowledge of the incorporation of other antimicrobial (AM) agents such as triclosan, cetylpyridinium chloride (CPC), and benzalkonium chloride (BC), along with their effects on cariogenic bacteria, the evidence remains unclear.^23-25^

 Cetylpyridinium chloride (C21H38NCl), an active ingredient in oral antiseptics, exhibits a wide antibacterial spectrum, demonstrating a potent bactericidal activity against gram-positive bacteria and a strong fungicidal effect. The efficacy against gram-negative bacteria and mycobacteria is uncertain. In comparison to chlorhexidine, CPC exhibits reduced residual effects, which consequently result in diminished efficacy against plaque and gingivitis. It can also be used as part of an oral healthcare regimen for patients wearing removable or fixed orthodontic appliances. The efficacy of CPC against oropharyngeal candidiasis has also been validated.^26-28^

 Benzalkonium chloride (alkyldimethylbenzylammonium chloride) is a potent biological agent with a moderate duration of action. This chemical exhibits activity against bacteria, some viruses, fungi, and protozoa. Bacterial spores are considered resistant. Solutions exhibit either bacteriostatic or bactericidal properties based on their concentration. Gram-positive bacteria exhibit greater sensitivity compared to gram-negative bacteria.^27,29^

 This study aimed to evaluate and compare the zones of inhibition of conventional GICs, with and without the addition of antimicrobial compounds CPC and BC, against cariogenic bacteria (*Streptococcus mutans, Lactobacillus casei*, and *Actinomyces viscosus*) across different time intervals.

## Hypothesis

- The initial hypothesis of the study posited that antibacterial GICs would markedly suppress caries-associated microorganisms (*S. mutans, L. casei*, and *A. viscosus*) in comparison to the group devoid of antibacterial agents.
- The second hypothesis was chosen based on the premise that the antibacterial activity may substantially diminish over time for either the control or experimental group.
- The third hypothesis posits that there is no statistically significant difference in the size of the inhibition zones between the two antimicrobial compounds.

## Methods

###  Materials

 In this study, the following materials were used: (1) commercially available conventional GICs, ChemFlex (DENTSPLY DeTrey, Konstanz, Germany) and Fuji IX (GC Int., Tokyo, Japan); (2) antimicrobial compounds: CPC produced by Sigma–Aldrich Co. under the trademark Cetylpyridinium Chloride C0732, and BC produced by Fluka Chemical Corporation, Milwaukee, WI, USA, under the trademark Benzalkonium Chloride 12660; (3) reference test strains of the following microorganisms: *S. mutans*(ATCC 25175), *L. casei* (ATCC4646), and *A. viscosus* (ATCC19246) in lyophilized form, manufactured by American Type Culture Collection, Manassas, VA, USA; (4) bacterial growth media (a) BHIB – Brain Heart Infusion Broth, Oxoid, Wesel, Germany, ready to use media in 10-mL test tubes; (b) Schaedler agar with the vaddition of sheep blood – Oxoid, Wesel, Germany, ready to use growth media in Petri dishes with a diameter of 90 mm; (5) anaerobic pots, with an atmosphere of 10% of carbon dioxide and 90% of nitrogen, employing an anaerobic system (Microbiology Anaerocult A, MERCK, Darmstadt, Germany).

###  Sample Preparation and Antimicrobial Agent Incorporation

**Figure 1:**
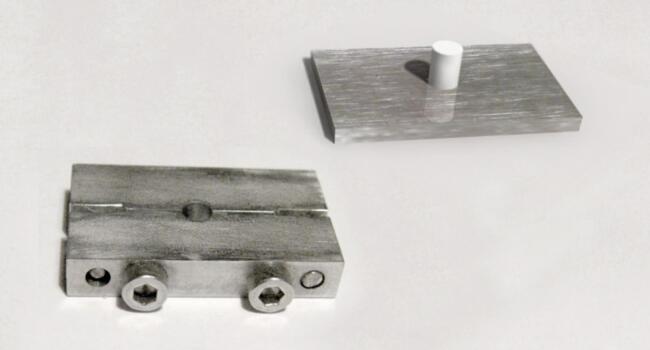
Metal mold and specimen of GIC

 Samples devoid of antimicrobial agents were prepared by combining a specified quantity of powder and liquid on glass mixing plates, following the manufacturer’s instructions. The newly mixed paste was thereafter placed into metal molds measuring 6 mm in height and 4 mm in diameter (Figure 1).

 The molds were enclosed with metal plates on both sides, fixed in specialized clamps, and incubated at 37 °C for one hour (maturation period). After being removed from the incubator, the specimens were withdrawn from the clamps and molds, and then individually preserved in labeled plastic tubes containing 5 mL of deionized water at 22‒24 °C and an air humidity of 40%‒50%.

 The AM agents CPC and BC were initially integrated into the polyacrylic acid of the GICs through manual mixing, followed by the gradual addition of the powder to the pre-prepared combination of acid and antimicrobial chemicals until saturation was achieved. The AM compounds have been incorporated in precise proportions of 1%, 2%, and 3% of the cement’s weight. The concentration (weight) of BC and CPC was determined using an analytical balance (Mettler AE 200). Previous analyses established that concentrations of 1%, 2%, and 3% of antimicrobial agents correspond to 0.0022, 0.0044, and 0.0066 g of the total cement mass of GIC ChemFlex, respectively, and 0.0032, 0.0064, and 0.0128 g of GIC Fuji IX. A total of 288 specimens were prepared, divided into four groups of 54 specimens each (comprising six specimens of GIC ChemFlex and six specimens of GIC Fuji IX, each incorporating three distinct concentrations of antimicrobial agents – CPC and BC) to assess the antimicrobial activity against three tested bacteria, alongside a control group of 72 specimens devoid of antimicrobial agents.

###  Microbiological Analysis

 The bacterial strains were inoculated into BHIB and incubated anaerobically at 37°C for 48 hours. The inoculum density derived from the bacteria cultured in the liquid medium was adjusted to align with the McFarland 2 standard. Subsequently, 350 μL of the bacterial suspension was uniformly distributed on the pre-marked Shaedler agar using a smear technique. After inoculation and an additional 15 minutes for the agar plate to absorb the bacterial suspension, sterile plastic tubes were used to create wells, measuring 4 mm in depth and 3.5 mm in width, in the agar plate. A total of 7 wells were prepared on each dish, 6 along the perimeter and one in the center. The distance between the wells was 30 mm, and the distance between the wells and the dish wall was 15 mm. On each dish, three specimens of GIC ChemFlex with 1%, 2%, and 3% CPC, respectively, and three samples with the corresponding percentages of BC were applied, along with one control sample (GIC without an antimicrobial compound). The same procedure was conducted for GIC Fuji IX.

**Figure 2:**
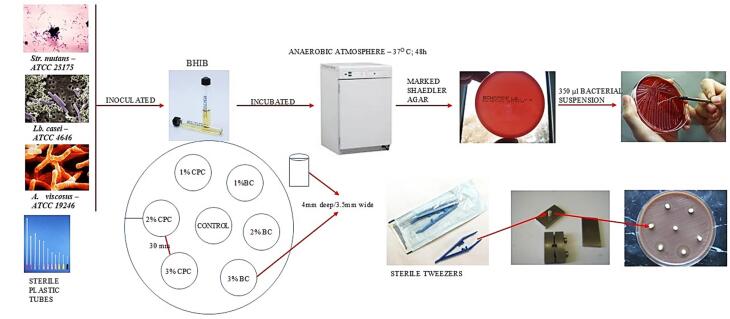
The complete procedures for microbiological analyses

 Under equal conditions, varying concentrations of each agent were compared with one another, and the effects of identical concentrations of two distinct agents were also analyzed. The specimens were carefully placed in the wells using sterile tweezers, ensuring they had close contact with the agar without tearing it. The Petri dishes were left at room temperature for 30 minutes and then incubated anaerobically at 37 °C for 48 hours. The initial measurement of the inhibitory impact, following a 48-hour incubation period, was designated as the effect at time zero. The analysis of the delayed effects of antimicrobial agents was undertaken after 2, 7, and 21 days. After each measurement, the Petri dishes were placed in a thermostat at 37 °C and maintained until the next measurement. Forty-eight hours before each subsequent measurement, identical specimens were consistently transferred from the thermostat to fresh spread growth media and incubated under identical conditions (anaerobically at 37 °C for 48 hours) until the intervals of 2, 7, and 21 days, at which point the inhibition zones were assessed and recorded (Figure 2).

###  Measurement of Inhibition Zones

**Figure 3:**
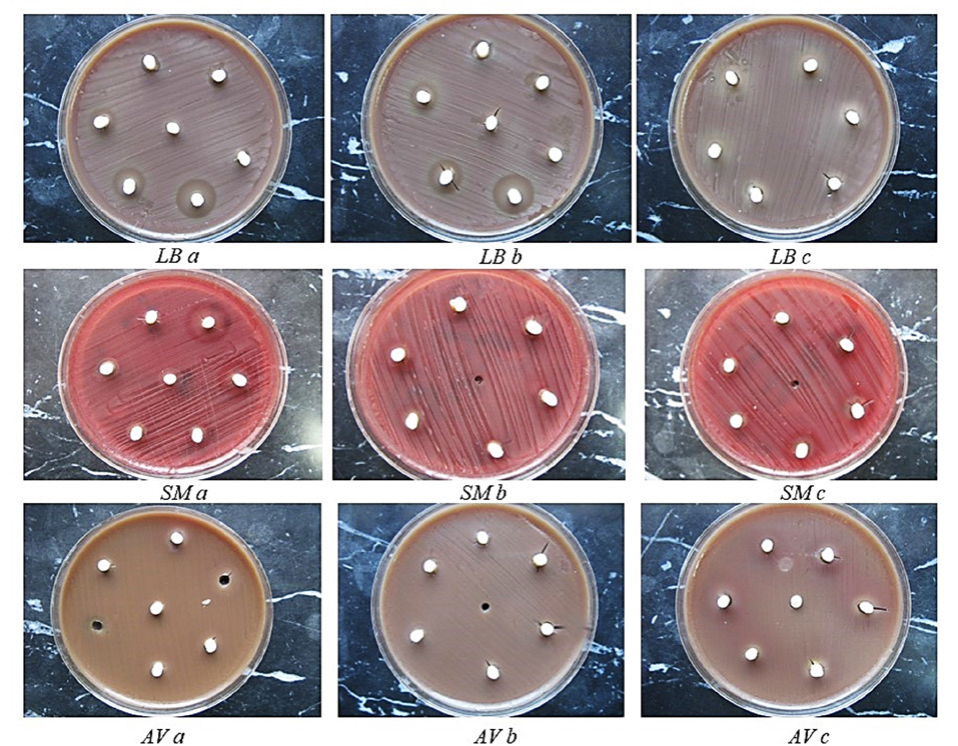
Petri dishes with the zone of inhibition formed around the specimens for *Lactobacillus casei (LB), Streptococcus mutans (SM), and Actinomyces viscosus (AV)*: (a) after 48h, (b) after 7d, (c) after 21d

 This continuous specimen preservation method replicated the conditions under which antimicrobial agents would operate in vivo, particularly within treated human teeth. The inhibitory zone was quantified by measuring its diameter in millimeters. The inhibitory zone’s size encompassed the diameter of the specimen (4 mm). The measurements for each specimen were conducted in two orthogonal directions. When the width of the inhibition zone fell between two integers, it was recorded to the nearest decimal point (Figure 3).

###  Statistical Analysis

 Statistical analyses were conducted using one-way ANOVA, followed by post hoc Tukey tests. The Statistica software was used for data processing.

## Results

Table 1 presents the average inhibition zones resulting from the combinations of GICs and antimicrobial agents against *S. mutans*. The elevation in AM compound concentration correlated with an expansion of inhibitory zones, with the most significant increase observed at a 3% concentration of AM compounds. The inhibitory zones diminished over time, reaching their minimum on the 21st day. The most extensive zones of inhibition were produced by the ChemFlex‒3% BC combination at both zero time and 48 hours (shown in Table 1 with §). The inhibition zones of the GIC/AM compound combinations were smaller than those of the control group in the combinations marked with ¶ in Table 1. The zones of inhibition decreased over time, reaching their lowest point at 21 days. Nevertheless, specific combinations of GICs and AM compounds demonstrated larger zones of inhibition in the later period compared to the early period, marked with ± in Table 1.

**Table 1:** Average inhibition zones of the antimicrobial agents on *Streptococcus mutans*

**Time**	**GIC+AM agents**	**0%** **Average (mm)±(SD)**	**1%** **Average (mm)±(SD)**	**2%** **Average (mm)±(SD)**	**3%** **Average (mm)±(SD)**
0h	ChemFlex + CPC	4.6 (0.49)	6.1 (0.92)	7.6 (1.02)	8.5 (0.77)
	ChemFlex + BC	4.6 (0.49)	11 (0.89)	13.3 (0.82)	14.9 (0.8)**§**
	Fuji IX + CPC	4.0 (0.0)	4.0 (0.0)	4.0 (0.0)	6.9 (1.09)
	Fuji IX + BC	4.0 (0.0)	4.0 (0.0)	4.0 (0.0)	4.5 (0.55)
48h	ChemFlex + CPC	4.7 (0.82)	4.9 (0.8)	6.5 (0.89)	7.4 (0.8)
	ChemFlex + BC	4.3 (0.41)	8.0 (0.0)	9.3 (0.52)	11.5 (1.22)**§**
	Fuji IX + CPC	4.3 (0.27)	4.0 (0.0)¶	4.0 (0.0)¶	5.1 (0.66)
	Fuji IX + BC	4.3 (0.26)	4.0 (0.0)¶	4.0 (0.0)¶	4.8 (0.93)**±**
7d	ChemFlex + CPC	4.5 (0.45)	4.0 (0.0)¶	5.0 (0.89)	7.2 (1.6)
	ChemFlex + BC	4.6 (0.38)	5.3 (0.41)	6.0 (1.1)	7.2 (1.17)
	Fuji IX + CPC	4.0 (0.0)	4.0 (0.0)	4.0 (0.0)	4.5 (0.84)
	Fuji IX + BC	4.6 (0.49)	4.0 (0.0)¶	4.0 (0.0)¶	4.0 (0.0)¶
21d	ChemFlex + CPC	4.5 (0.45)	4.0 (0.0)¶	4.7 (0.82)	6.5 (0.84)
	ChemFlex + BC	4.4 (0.49)	4.7 (0.82)	5.3 (1.63)	6.3 (2.66)
	Fuji IX + CPC	4.3 (0.27)	4.0 (0.0)¶	4.0 (0.0)¶	5.7 (1.86)**±**
	Fuji IX + BC	4.3 (0.41)	4.5 (0.55)**±**	5.3 (1.51)**±**	7.7 (4.03)**±**
mean value			5,03	5,7	7,04

¶ Inhibition zones smaller than the control. **§** The largest inhibition zones. **±**Bigger inhibition zones than in previous periods.

 One-way ANOVA, followed by post hoc Tukey tests, indicated that the differences in mean inhibition values for *S. mutans* were significant for all combinations, except the Fuji IX + CPC combination on the seventh day, the ChemFlex + BC, and the Fuji IX + BC combinations on the 21st day (Table 2).

**Table 2:** Average inhibition zones of the antimicrobial agents on *Lactobacillus casei*

**Time**	**GIC+AM agents**	**0%** **Average (mm)±(SD)**	**1%** **Average (mm)±(SD)**	**2%** **Average (mm)±(SD)**	**3%** **Average (mm)±(SD)**
0h	ChemFlex + CPC	5.0 (1.1)	4.3 (2.21)	5.9 (0.8)	6.7 (0.26)
	ChemFlex + BC	5.0 (1.1)	7.1 (0.66)	9.6 (1.28)	11.8 (1.33)**§**
	Fuji IX + CPC	4.0 (0.0)	5.1 (1.28)	6.2 (1.7)	7.3 (0.67)
	Fuji IX + BC	4.0 (0.0)	5.9 (1.28)	8.9 (2.15)	13.0 (1.9)**§**
48h	ChemFlex + CPC	4.4 (0.49)	4.1 (0.2)¶	4.5 (0.63)	6.0 (0.0)
	ChemFlex + BC	4.6 (0.49)	5.9 (0.49)	7.3 (0.69)	8.8 (0.98)
	Fuji IX + CPC	4.1 (0.2)	4.3 (0.82)	5.2 (0.98)	6.6 (0.49)
	Fuji IX + BC	3.9 (0.38)	4.3 (0.52)	6.5 (0.52)	9.8 (1.47)
7d	ChemFlex + CPC	5.0 (0.55)	4.0 (0.0)¶	4.0 (0.0)¶	5.8 (0.75)
	ChemFlex + BC	4.6 (0.80)	5.1 (0.92)	6.5 (0.84)	7.7 (0.82)
	Fuji IX + CPC	4.3 (0.27)	4.0 (0.0)¶	5.0 (0.89)	6.4 (0.49)
	Fuji IX + BC	4.5 (0.45)	4.0 (0.0)¶	5.5 (1.05)	8.7 (2.73)
21d	ChemFlex + CPC	5.0 (0.89)	4.0 (0.0)¶	4.0 (0.0)¶	5.3 (0.52)
	ChemFlex + BC	4.8 (0.93)	4.5 (0.55)¶	6.0 (0.63)	7.5 (0.55)
	Fuji IX + CPC	4.0 (0.0)	4.0 (0.0)¶	4.5 (0.55)	5.3 (1.5)
	Fuji IX + BC	4.3 (0.27)	4.0 (0.0)¶	6.2 (2.04)**±**	9.2 (2.48)**±**
mean value			4,7	6	7,9

¶ Inhibition zones smaller than the control. **§** The largest inhibition zones. **±**Bigger inhibition zones than in previous periods.

Table 3 presents the average inhibition zones resulting from the combinations of GICs and AM agents against *L. casei*. The rise in AM compound concentration correlated with an increase in inhibitory zones, with the maximum observed at 3% concentration. The inhibitory zones declined over time, reaching their minimum on the 21st day. The most extensive zones of inhibition were produced by the ChemFlex‒3% BC combination and the Fuji IX‒3% BC combination, both at zero time, as indicated in Table 1 with §. The inhibition zones of the GIC/AM compound combinations were inferior to the control group in the combinations marked with ¶ in Table 3. The inhibitory effect of combinations of GICs/AM compounds decreased over time, reaching its lowest point on day 21 of the analysis. Nonetheless, the Fuji IX + BC 2% and 3% combination on day 21 produced bigger zones of inhibition compared to earlier examined intervals (marked with ± in Table 3).

**Table 3:** Average inhibition zones of the antimicrobial agents on *Actinomyces viscosus*

**Time**	**GIC+AM agents**	**0%** **Average (mm)±(SD)**	**1%** **Average (mm)±(SD)**	**2%** **Average (mm)±(SD)**	**3%** **Average (mm)±(SD)**
0h	ChemFlex + CPC	4.2 (0.26)	6.4 (1.2)	8.1 (1.35)	9 (1.3)
	ChemFlex + BC	4.2 (0.26)	7.8 (1.17)	10.6 (1.2)	11.8 (1.33)**§**
	Fuji IX + CPC	4.1 (0.2)	4.6 (0.66)	5.8 (0.61)	7.6 (0.5)
	Fuji IX + BC	4.1 (0.2)	6.6 (0.57)	8.3 (1.08)	11.8 (0.75)**§**
48h	ChemFlex + CPC	4.5 (0.44)	6.8 (1.33)**±**	8.6 (2.25)**±**	10.3 (3.4)**±**
	ChemFlex + BC	4.8 (0.75)	6.6 (1.3)	8.8 (2.36)	10.6 (3.83)
	Fuji IX + CPC	4.1 (0.2)	4.0 (0.0)¶	5.2 (0.75)	6.7 (0.42)
	Fuji IX + BC	4.1 (0.2)	4.0 (0.0)¶	6.6 (0.5)	9.7 (1.03)
7d	ChemFlex + CPC	5.2 (0.7)	5.1 (0.8)¶	6.1 (0.8)	7.3 (1.03)
	ChemFlex + BC	4.8 (0.75)	5.3 (0.82)	6.8 (1.7)	8.5 (2.07)
	Fuji IX + CPC	4.3 (0.27)	4.2 (0.41)¶	5.4 (1.02)**±**	7.3 (1.17)**±**
	Fuji IX + BC	4.2 (0.26)	4.0 (0.0)¶	5,0 (0,0)	7.0 (0.84)
21d	ChemFlex + CPC	4.5 (0.87)	4.3 (0.82)¶	5.2 (0.98)	6.2 (0.98)
	ChemFlex + BC	4.0 (0.0)	5.8 (2.04)	7.3 (2.66)**±**	9.3 (3.01)**±**
	Fuji IX + CPC	4.0 (0.0)	4.0 (0.0)¶	4.7 (0.82)	6.8 (0.75)
	Fuji IX + BC	4.3 (0.41)	4.0 (0.0)¶	4.5 (0.55)	6.7 (0.52)
mean value			5.2	6.7	8.5

¶ Inhibition zones smaller than the control. **§** The largest inhibition zones. **±**Bigger inhibition zones than in previous periods.

 The mean inhibitory values for *L. casei* across all combinations of GICs/AM agents differed statistically, as determined by one-way ANOVA followed by post hoc Tukey tests.

Table 4 presents the mean zones of inhibition for the combinations of GICs/AM agents against *A. viscosus*. The most extensive zones of inhibition were observed in the ChemFlex‒3% BC and Fuji IX‒3% BC combinations at zero time (shown by § in Table 4). As the concentration increased, the zones of inhibition expanded, with all combinations exhibiting the maximum effect upon the incorporation of 3% AM agent. The combinations marked with ¶ in Table 4 demonstrate reduced zones of inhibition relative to the control group. The zones of inhibition decreased over time, reaching their minimum at day 21. However, the combinations marked with ± in Table 4 exhibit bigger zones of inhibition in the later period compared to the early period. One-way ANOVA, followed by post hoc Tukey tests, indicated that the mean inhibition values for *A. viscosus* were significantly different across all combinations of GICs/AM agents (Table 2).

**Table 4:** Statistical significance (*P* < 0,05) obtained by One-way ANOVA followed by post hoc Tukey tests for each combination of GIC/AM agents against cariogenic microorganisms

***P*** ** value**	**Time**	**ChemFlex+BC**	**ChemFlex+CPC**	**Fuji IX+BC**	**Fuji IX+CPC**
*Streptococcus mutans*	0h	0.000000	0.000000	0.009510	0.000003
	48h	0.000000	0.000024	0.022308	0.000090
	7d	0.000234	0.000059	0.000798	0.126738*
	21d	0.208924*	0.000006	0.055932*	0.016443
*Lactobacillus casei*	0h	0.000000	0.023282	0.000000	0.000044
	48h	0.000000	0.000000	0.000000	0.000016
	7d	0.000013	0.000002	0.000102	0.000001
	21d	0.000001	0.000216	0.000058	0.028761
*Actinomyces viscosus*	0h	0.000000	0.000002	0.000000	0.000000
	48h	0.002001	0.001096	0.000000	0.000000
	7d	0.000418	0.000455	0.000000	0.000005
	21d	0.004191	0.009255	0.000000	0.000000

*Statistically not significant at *P >*0.05.

 Statistical analyses using one-way ANOVA, followed by post hoc Tukey tests for various combinations of GIC/AM agents at each concentration, are detailed in Table 5. In the control group, specifically in GICs without AM agents, there was no significant difference between the three microorganisms. In contrast, at all concentrations (1%, 2%, and 3%), the differences were significant, except on the 21st day for *S. mutans* across all three concentrations, and on the 48th hour and the 7th day for *A. viscosus* at 3% antimicrobial compounds.

**Table 5:** Statistical significance (*P* < 0.05) obtained by one-way ANOVA followed by post hoc Tukey tests between combinations of GIC/AM agents for each concentration

***P*** ** value**	**Time**	**0%** **ChemFlex+CPC** **ChemFlex+BC** **Fuji IX+CPC** **Fiji IX+BC**	**1%** **ChemFlex+CPC** **ChemFlex+BC** **Fuji IX+CPC** **Fiji IX+BC**	**2%** **ChemFlex+CPC** **ChemFlex+BC** **Fuji IX+CPC** **Fiji IX+BC**	**3%** **ChemFlex+CPC** **ChemFlex+BC** **Fuji IX+CPC** **Fiji IX+BC**
*Streptococcus mutans*	0h	0.005766	0.000000	0.000000	0.000000
	48h	0.487665*	0.000000	0.000000	0.000000
	7d	0.043884*	0.000000	0.000176	0.000025
	21d	0.749978*	0.058568*	0.189905*	0.619987*
*Lactobacillus casei*	0h	0.040182	0.020541	0.000279	0.000000
	48h	0.040182	0.000038	0.000484	0.000001
	7d	0.159225*	0.000841	0.000309	0.016615
	21d	0.050580*	0.009510	0.005252	0.000488
*Actinomyces viscosus*	0h	0.855858*	0.000086	0.000003	0.000001
	48h	0.072388*	0.000181	0.003836	0.069182*
	7d	0.012478	0.001950	0.009033	0.261147*
	21d	0.192917*	0.026229	0.013577	0.015150

*Statistically not significant at *P* > 0.05.

**Figure 4:**
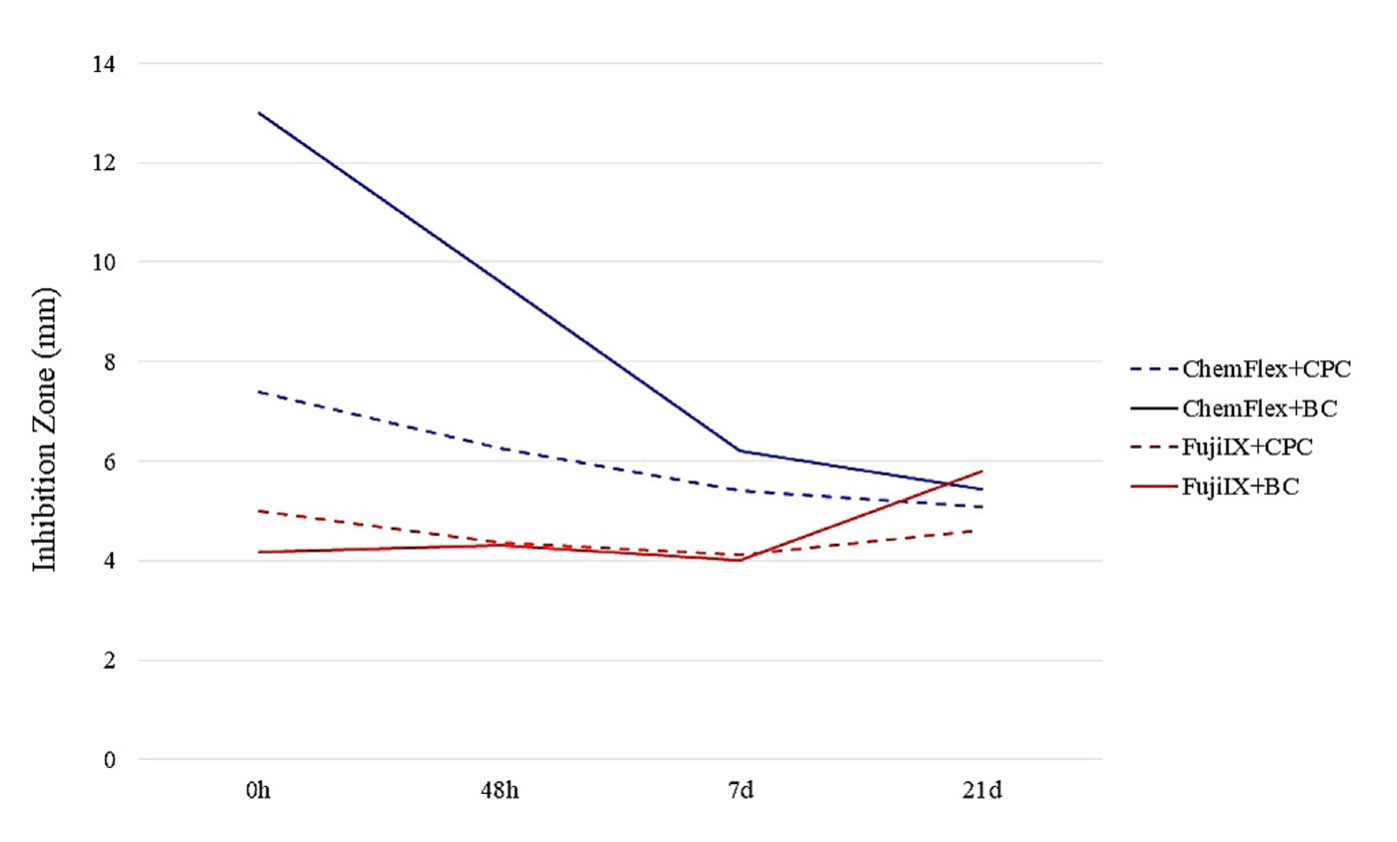
The overall average effects of all combinations on *Streptococcus mutans*

**Figure 5:**
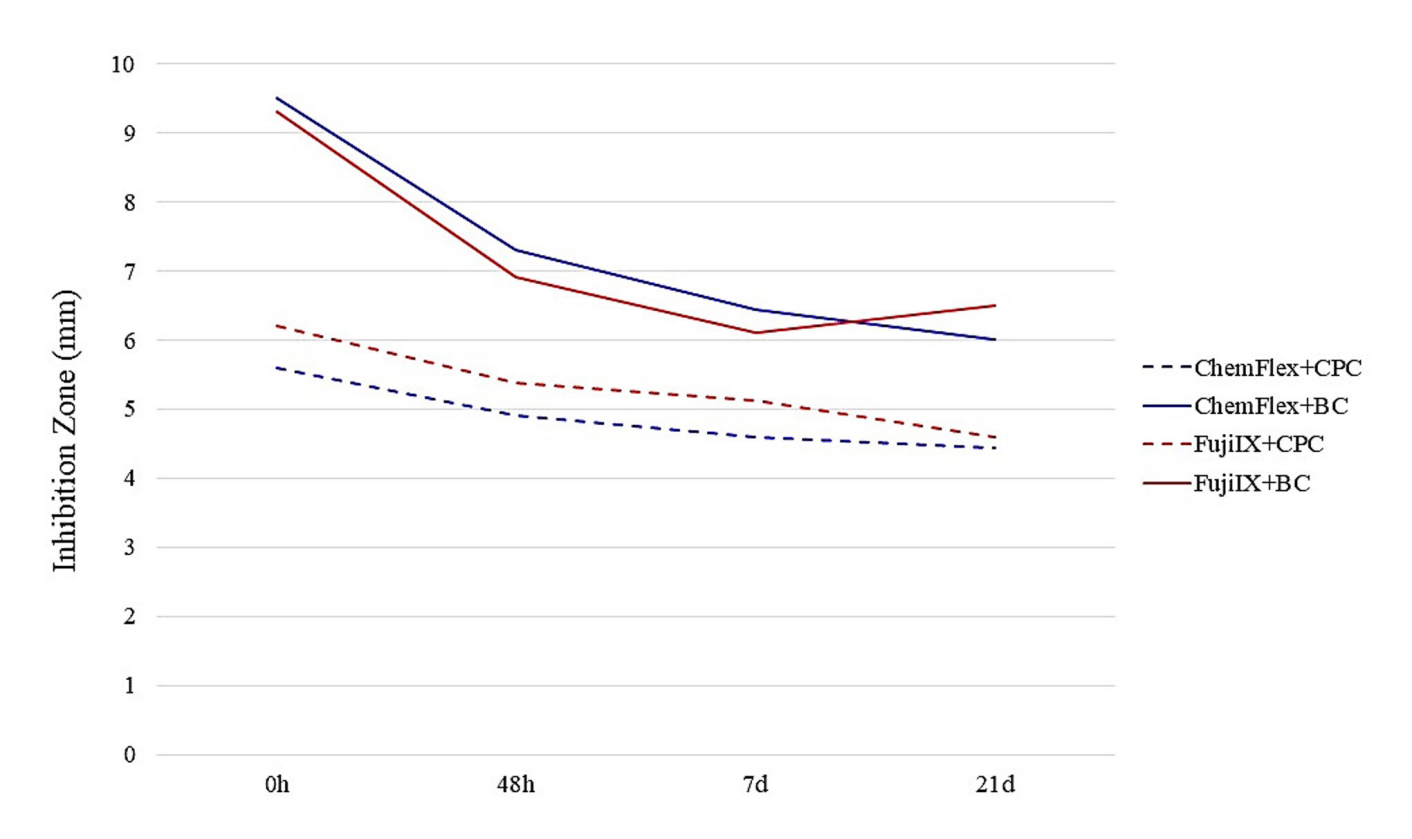
The overall average effects of all combinations on *Lactobacillus casei*

**Figure 6:**
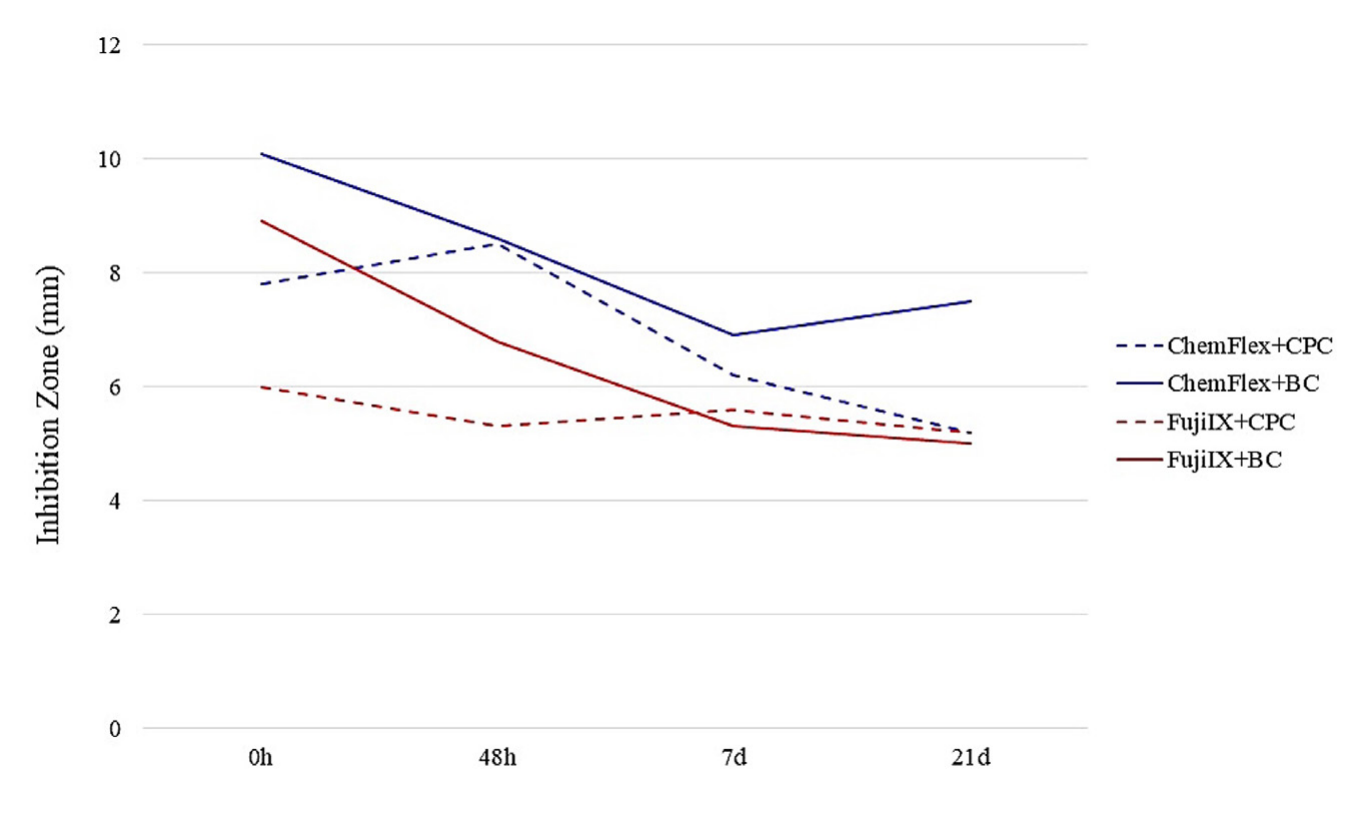
The overall average effects of all combinations on *Actinomyces viscosus*

 Summary graphs were created to provide a precise insight into the suppressive action of the GIC/AM compound combinations on each of the examined bacteria. Figures 4, 5, and 6 present the total mean values of GIC/AM compounds on cariogenic microorganisms. These values were derived from the mean zones of inhibition for each concentration (1%, 2%, and 3%), considering the cross-sectional combination and each time interval.

**Figure 7:**
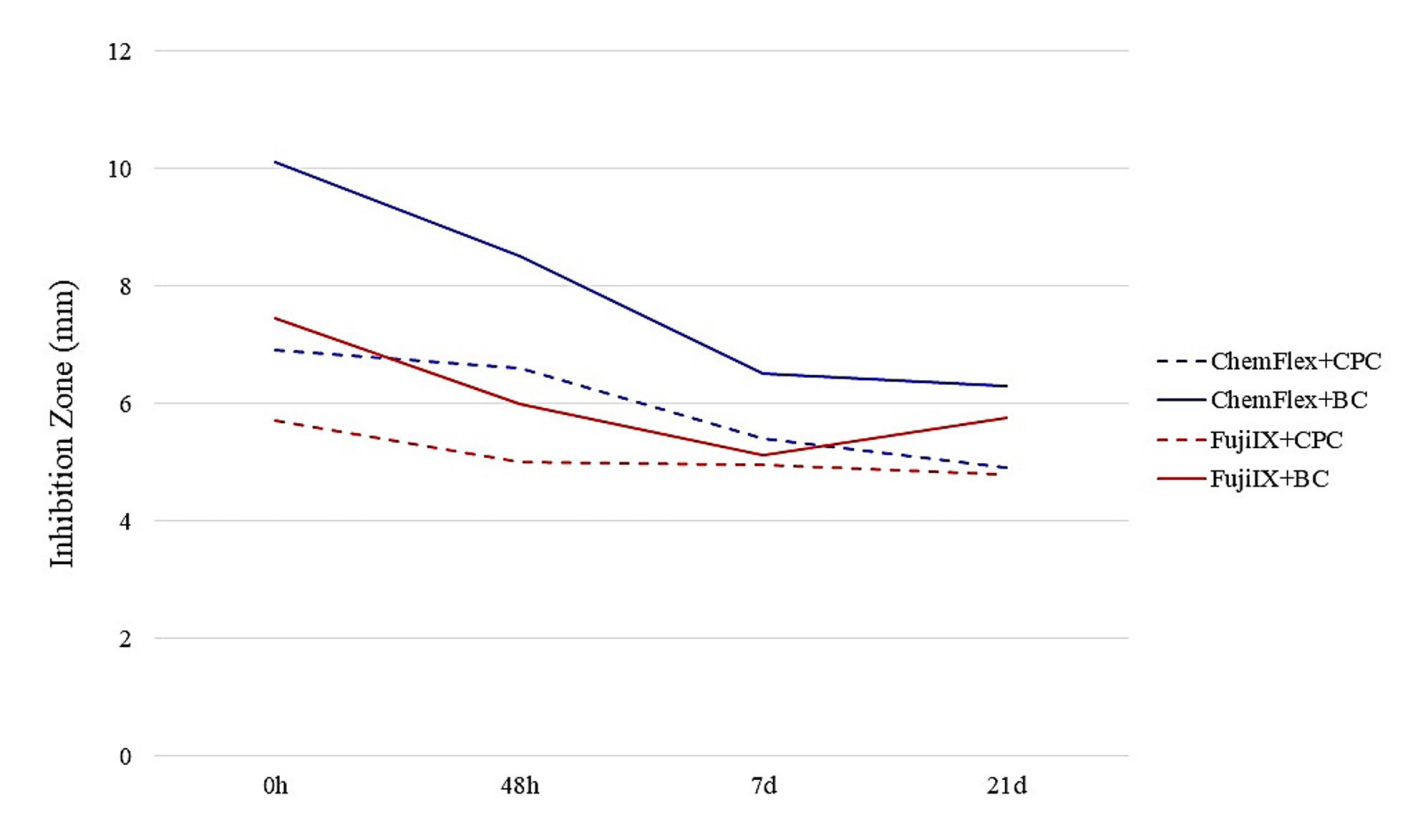
The overall effect of GICs and antimicrobials on cariogenic microorganisms

Figure 7 illustrates the cumulative antimicrobial efficacy of the four GIC/AM compound combinations at three concentrations, assessed against all tested microorganisms. These data were derived from the previously calculated average inhibition zone values for each GIC/AM compound combination, within the designated time frame, and for each bacterial strain.

## Discussion

 Microbiological analyses were conducted on reference cariogenic microorganisms to assess the antimicrobial properties of GICs containing AM compounds. These microorganisms are found at various locations on the dental surface and are the primary pathogens that contribute to the occurrence and development of dental caries.

###  Previous Findings, Types of AM Agents, Methods of Incorporation, and Microbiological Analyses

 While microbiological analyses can theoretically use saliva, plaque isolates, or carious lesions from a cohort, leading to the isolation of various microorganism subtypes, the literature predominantly relies on laboratory-derived reference species. This approach ensures that the results have an international reference value. The reference strains of the cariogenic bacteria *S. mutans, L. casei*, and *A. viscosus* have been thoroughly analyzed.^1,2,8,10,18-20,22-24,30,31^

 The antibacterial efficacy of specific substances or agents can be assessed using various microbiological analyses and procedures. The most commonly used microbiological method is the agar diffusion test, and the inhibition zones are determined in millimeters.^17,19,20,23,24,29,32-34^ Furthermore, zones of inhibition, reflecting the cumulative effects of the compounds, may also be expressed as areas of inhibition (mm^2^).^29,35^ The shape and the way of placement of the cement samples on the Petri dishes are an issue. Globally, there are two ways of placement: preparation of wells of certain dimensions in the agar and placement of the freshly mixed cement (the so-called unset cement),^17,19,20,23,35,36^ and preparation of samples of the cement, its setting and maturation in laboratory conditions, and placement of the samples in the previously prepared wells in the agar (set cement).^19,24,36,38^

 The incubation time of the Petri dishes varies as well. In some studies, it was 24 hours, and in most of them, it was 48 hours, which is, in principle, a better solution for the higher growth of the inoculated bacteria.^20,23,24,33,35,37,38^ The sizes of the samples of the set cement vary among authors, which has influenced the size of the inhibition zones. In addition to the 6 × 4-mm samples for microbiological analysis, 10 × 2-mm, 10 × 4-mm, or 5 × 2-mm samples are also prepared. However, when calculating the size of the inhibition zones, the size of the sample must always be taken into account.^17,20,23,24,35^

 The majority of research regarding the incorporation of AM agents into GICs has focused on chlorhexidine in various forms, including diacetate, digluconate, gluconate, or hydrochloride, and its antibacterial effects on cariogenic microorganisms have been extensively examined.^19-22,32,33^ Certain investigations indicate a more significant antibacterial efficacy of embedded versions of CHX compared to other embedded antimicrobial agents or products (cytosine, propolis, and cetrimide).^17,19,20^ Nonetheless, some studies indicate that CHX exhibits weaker antibacterial efficacy compared to other evaluated antimicrobials, such as BC, CPC, or triclosan.^19,29^

###  Analysis of the Results and Comparison with Existing Literature 

 This study aimed to assess the antimicrobial efficacy of the quaternary ammonium compounds CPC and BC against reference strains of prevalent cariogenic microorganisms, using the agar diffusion method with sample dimensions of 6 × 4 mm across four time intervals. Numerous studies have indicated that increased concentrations lead to larger inhibition zones; however, these zones frequently decrease over time.^19-21,24,33,35^ However, there are also some opposing opinions, i.e., that the inhibition zones are not dependent on the concentration or the type of incorporated compound.^32,37^ According to the results of the present study, AM GICs produced significantly larger inhibition zones, effectively suppressing caries-associated microorganisms in correlation with increasing concentrations of AM compounds compared to the control group. However, exceptions were observed for specific combinations at certain intervals involving the addition of 1% and 2% antimicrobial agents, indicating partial confirmation of the initial hypothesis.

 Although the zones of inhibition for some combinations were larger in the later periods, the antimicrobial activity of the experimental group decreased over time, leading to reduced zones of inhibition as time progressed, confirming the second hypothesis.

 While a recognized antimicrobial impact exists in mouthwashes and certain medical lozenges, there is a paucity of studies detailing the effects of CPC and BC when integrated into GICs. The statistical analysis of the antimicrobial effects between the two compounds indicated no significant differences in the control group. Statistically significant differences were noted in the experimental group, except on the 21st day for *S. mutans* across all concentrations, and the 48th hour and 7th day for *A. viscosus* in 3% AM agents. The results partially confirmed the third hypothesis. This study’s evaluation of the inhibitory effects of GIC containing BC and CPC on cariogenic bacteria is consistent with specific findings in the literature.^24,35^

 The literature presents varying data regarding which cariogenic bacteria exhibit a more pronounced antimicrobial response to the embedded agents in GICs. Their dependency fundamentally hinges on the type of agent, whether used singularly or in conjunction with another agent (binary action), but predominantly on the concentration. Certain studies suggest that the antimicrobial activity was more evident against *S. mutans* in comparison to *Lactobacillus *species,^17,23^ while other research indicated that the activity was more substantial against *Lactobacillus *species.^19,20,22,23,36^ The antimicrobial activity against* A. viscosus* was weaker than or equal to that observed for the previous two bacteria in the comparative analyses.^23,36^ In contrast to the literature data, our investigation reveals that the average inhibitory zone values corresponding to the concentration of AM compounds showed that the most significant decrease occurred with 1% AM compounds against *A. viscosus*. In contrast, the least reduction was observed against *L. casei*. The most significant decreases were reported with the 2% and 3% AM compounds against *A. viscosus*, followed by *L. casei*, while the least reduction occurred against *S. mutans*.

 Although the average values of the inhibition zones support *A. viscosus*, individually, the largest zones of inhibition were obtained from the ChemFlex‒3% BC combination at zero hour against *S. mutans*, and from the Fuji IX‒3% BC combination likewise in the same timeframe against *L. casei*. The combination of ChemFlex with BC exhibited superior efficacy against all three microorganisms. In the comparison of the antibacterial efficacy of the AM agents, the compound BC exhibited a greater effect.

 The possibility of incorporating CPC and BC into GICs, even at a concentration of 1%, would significantly enhance the antimicrobial effect of the cements, leading to a substantial reduction in cariogenic flora.

###  Interpretation of Unexpected Results

 Opinions vary about the superiority of various methods for analyzing antibacterial characteristics. The assertion that unset cements produce larger inhibitory zones has been validated, which is rational due to the considerable mobility of the molecules in their liquid state.^36,37^ In any case, if the antimicrobial effect of unset materials is to be determined, an analysis should also be carried out with samples prepared from the same materials under the same working conditions.

 The observed inhibitory zones in the control group are likely attributable to the inherent antibacterial characteristics of the cements due to the action of fluorides, a constituent of GICs, whose antibacterial capabilities have been validated. It is noteworthy that in specific combinations of GIC/AM compound, the inhibitory zones, notably with the 1% AM compounds, are smaller than those of the control group. A plausible explanation for this phenomenon, linked to the manual mixing of cement with AM compounds, may be the “delayed burst effect,” resulting from inadequate distribution and “trapping” of the AM compound molecules, which could lead to increased retention of these molecules in the depths of the samples. The low concentration prevents the molecules from penetrating the sample’s surface and thus releasing themselves promptly within the created cement. The manual fabrication of GIC samples in this study could be enhanced by modifying the sample preparation process to incorporate mechanical mixing of the components. Furthermore, the development of bigger zones of inhibition in subsequent periods compared to the initial time for specific combinations of GICs/AM compounds is considered a paradox. The quick release of a greater volume of previously “trapped” molecules of the AMs within the samples may clarify these events, as they gradually “ascend” to the surface. Further analyses are necessary to support these observations.

## Conclusion

 The incorporation of AM agents CPC and BC into conventional GICs—ChemFlex and Fuji IX—exhibits an inhibitory effect on cariogenic bacteria *S. mutans, L. casei*, and *A. viscosus*. This effect is more pronounced at elevated concentrations. Throughout subsequent periods, the suppressive effect decreased while it remained markedly potent. GICs devoid of AM agents exhibited a limited yet discernible antibacterial activity. The future development of antimicrobial glass ionomer cements represents a significant benefit in the fight against dental caries.

## Competing Interests

 The authors declare no competing interests. The authors certify that they have no affiliations with or involvement in any organization or entity with any financial interest (such as honoraria; educational grants; participation in speakers’ bureaus; membership, employment, consultancies, stock ownership, or other equity interest; and expert testimony or patent-licensing arrangements), or non-financial interest (such as personal or professional relationships, affiliations, knowledge or beliefs) in the subject matter or materials discussed in this manuscript.

## Ethical Approval

 This study was approved by the Research Ethics Committee, Faculty of Dental Medicine, Ss Cyril and Methodius University in Skopje, Republic of North Macedonia, under protocol number 09 – 1353/1 on 24.04.2025.
